# Effect of knowledge of informal poultry drug prescribers on their attitude and practice toward antimicrobial use, residues, and resistance in Bangladesh

**DOI:** 10.14202/vetworld.2023.1821-1828

**Published:** 2023-09-14

**Authors:** Aminatu Abubakar Sani, Kazi Rafiq, Fatema Akter, Purba Islam, Sabbya Sachi, Nasrin Sultana, Sajedul Hayat, Usman Bashir Usman, Md. Shafiqul Islam, Md. Zahorul Islam, Muhammad Tofazzal Hossain

**Affiliations:** 1 Department of Pharmacology, Bangladesh Agricultural University, Mymensingh, Bangladesh; 2 Department of Pharmacology and Toxicology, Faculty of Veterinary Medicine, Usmanu Danfodiyo University, Sokoto, Nigeria; 3 Department of Anatomy and Histology, Bangladesh Agricultural University, Mymensingh, Bangladesh; 4 Department of Veterinary Public Health and Preventive Medicine, Faculty of Veterinary Medicine, Ahmadu Bello University Zaria, Nigeria; 5 Department of Microbiology and Hygiene, Bangladesh Agricultural University, Mymensingh, Bangladesh

**Keywords:** antimicrobial residue, antimicrobial resistance, antimicrobial use, informal poultry drug prescriber, knowledge, attitude, and practices, survey

## Abstract

**Background and Aim:**

Informal prescribers (IPs) significantly contribute to the development of antimicrobial resistance and in disseminating pathogens from poultry to humans and other animals through the food chain, posing a serious global health threat. Therefore, this study aimed to assess whether the knowledge of IPs has an impact on their attitude and practice toward antimicrobial use, antibiotic residues, and antimicrobial resistance.

**Materials and Methods:**

In this cross-sectional study, we conducted a pre-tested and questionnaire-based survey to investigate the knowledge, attitude, and practice of IPs in selected parts of the Mymensingh division, Bangladesh. Then, we used the linear regression model test with R-squared (R^2^) to measure the association between the study variables.

**Results:**

Our investigation revealed that 70% of the IPs knew about antibiotics and 75% had good knowledge about antibiotic resistance, whereas only 50% were aware of withdrawal periods. Informal prescribers also displayed good attitudes toward the use and sale of antibiotics with withdrawal periods and completion of medication (50%). Analysis of their practice on the sale and prescription of antibiotics showed that 70% and 30% of IPs use antibiotics against bacterial infections and other conditions, respectively. Most of them do not consult a veterinarian before selling or prescribing antibiotics, although 80% claim to do so. This is because 75% of IPs gave other options regarding their consultations. However, 95% of IPs uses antibiotics only for therapeutic purposes. Furthermore, only 10% sell antibiotics based on a veterinarian’s recommendation. Approximately 45% of IPs use single antibiotics at a time, while the rest use multiple antibiotics, individually or combined. Approximately 15% use antibiotics monthly, while 85% use them whenever the need arises. The knowledge and attitude of IPs are significantly affected by their age (p ≤ 0.025). The district of domicile also impacted their knowledge. Surprisingly, IPs from Jamalpur had significantly better knowledge compared to those from Mymensingh and Sherpur (p ≤ 0.01). The attitude of IPs from Jamalpur and Netrokona also differed significantly (p ≤ 0.001) from that of Mymensingh and Sherpur. The knowledge of IPs influenced their attitude up to 80.5% (r^2^ = 0.628) and their practice up to 75.4% (r^2^ = 0.545).

**Conclusion:**

The knowledge of IPs greatly influenced their attitude and practice, while sociodemographics also influenced their knowledge and attitude toward antimicrobial use, antibiotic residues, and antimicrobial resistance.

## Introduction

Informal prescribers (IPs) are involved in poultry and larger livestock industries as they act as the midpoint between the sale and administration of antibiotics and other important drugs in the public health sector. However, disturbingly, many of them lack adequate knowledge regarding the proper use of drugs and the associated challenges in the health sector. The antimicrobials that are generally used for treatment and prevention are also incorporated into the production as “growth promoters” and increase the feed conversion ratio [1]. Even for the educated, the rationale use of drugs only seems theoretical because they believe that the demand justifies the breach of protocol. Although antimicrobials are important for maintaining healthy poultry production, non-judicious use can cause undesirable issues, such as antimicrobial resistance (AMR) [2–4].

In Bangladesh, the production of poultry meat and eggs has risen due to increased demand, making this a profitable sector. However, this growth is accompanied by a high level of antibiotic use, though the statistics are unknown [5]. The data available on informal drug sales are inadequate and unreliable [6]. Moreover, antimicrobials are mostly sold by company representatives, traders, and feed sellers, all under the umbrella of “informal prescription” from the point of view of professionals [7]. However, the role of IPs cannot be ignored because the number of field veterinarians in Bangladesh is insufficient to meet the demands [8]. Furthermore, small-scale farmers are even provided with soft loans by some IPs, which indirectly puts them under the control of these prescribers, for example, feed dealers [8]. The antimicrobials used in the livestock industry are mostly obtained from the IPs’ chain of distribution or agro-veterinary shops where over half of the owners do not possess the necessary qualifications to do so in the developing world [9]. Inadequate knowledge, attitude, and practice (KAP) have also been documented to affect antimicrobial use (AMU) and AMR, respectively, among many livestock-rearing communities in the developing world [10]. Improper prescription of antibiotics by IPs significantly increases the risk of AMR among livestock and subsequently, the public health sector.

The lack of proper knowledge on the use and misuse of antimicrobials affects the attitude and practice of the sale and prescription of drugs by IPs. Although AMR is a significant global health threat, the data on the mechanism and the players involved in this crisis are insufficient. Therefore, further data on the IPs’ KAP related to AMU and AMR are required. This could provide crucial information that can help control AMR. This study aimed to assess the knowledge of the IPs and their effect on their attitude and practice of AMU, AMR, and antimicrobial residues. This will supplement the information on the contribution of the IPs to the sale and prescription of medicines.

## Materials and Methods

### Ethical approval and Informed consent

The project protocol was approved by the Animal Welfare and Experimentation Ethics Committee of Bangladesh Agricultural University, Mymensingh (approval number: AWEEC/BAU/2018(31); date: December 30, 2018). The verbal consent was obtained from the participants.

### Study period and location

The study was conducted from June to December 2019 in four districts of Mymensingh division, Bangladesh.

### Study design

This is a cross-sectional study in which a pre-tested questionnaire-based survey was conducted to investigate the KAP of 20 IPs in select parts of the Mymensingh division, Bangladesh (Table-1). We selected the interviewees from four districts in the Mymensingh division based on their availability during the interview.

**Table-1:** Sociodemographic characteristics of IPs.

Characteristics	Category	Frequency (Number of IPs)	Percentage
Age	25–35	6	30.0
	35–45	3	15.0
	45–55	5	25.0
	55–65	6	30.0
Education	Secondary (SSC)	3	15.0
	HSC/Diploma	6	30.0
	Graduate	5	25.0
	Masters	6	30.0
Occupation	Veterinary field staff	7	35.0
	Veterinary compounder	1	5.0
	Village animal doctor/quack	5	25.0
	Veterinary drug seller	4	20.0
	Medicine company professional	3	15.0
Duration of practice	1 year	1	5.0
	2 years	1	5.0
	Others	18	90.0

IPs=Informal prescribers

### Data collection and questionnaire survey

**Figure-1:**
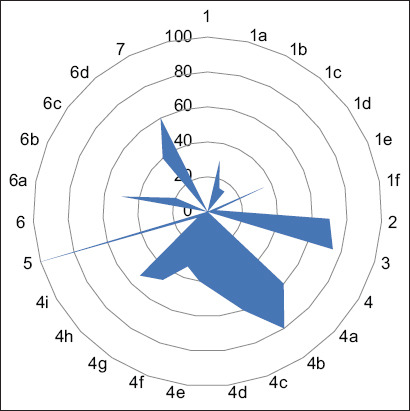
Response on knowledge of IPs on AMU and AMR. Question 1: Do you have knowledge about antibiotics? (Q1a) Antibiotics act against bacteria? (Q1b) Antibiotics act against virus? (Q1c) Others? (Q1d) Don’t know? (1Qe) Antibiotics act against bacteria and virus? (Q1f) Antibiotics act against bacteria, virus, and others? (Yes/No). Question 2: Do you know about withdrawal period of antibiotics? (Yes/No). Question 3: Heard about antibiotic resistance? (Yes/No). Question 4: What are the causes of resistance? (Q4a) Incomplete antibiotic course? (Q4b) Using the same antibiotic frequently? (Q4c) Overdose? (Q4d) Low dose? (Q4e) Skipping dose? (Q4f) Taking different antibiotics same time? (Q4g) Taking antibiotics that have been kept long time? (Q4h) Adulterated antibiotics? (Q4i) Others? (Yes/No). Question 5: Have knowledge about antibiotic resistance? (Yes/No). Question 6: What do you know about antibiotic resistance? (Q6a) It causes treatment failure? (Q6b) It causes poor response to treatment? (Q6c) Do not know? (Q6d); Others? (Yes/No). Question 7: Have knowledge about antibiotic residues? (Yes/No). IPs=Informal prescribers, AMU=Antimicrobial use, AMR=Antimicrobial resistance.

**Figure-2:**
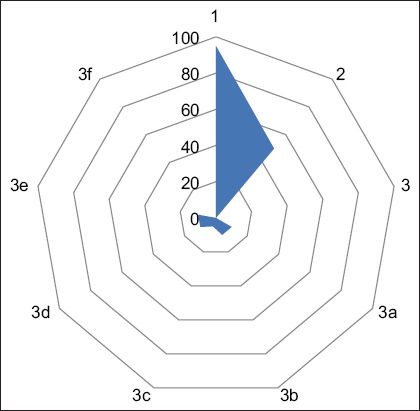
Response on attitude of IPs on AMU and AMR. Question 1: Do you suggest for completion of antibiotic course? Yes/No. Question 2: Do you advise farmers to follow withdrawal period? (Yes/No). Question 3: Sell of antibiotic recommended by? (3a) veterinarian; (3b) other farmers; (3c) pharmaceutical company representative; (3d) village doctor; (3e) quack; (3f) all of the above? (Yes/No). IPs=Informal prescribers, AMU=Antimicrobial use, AMR=Antimicrobial resistance.

**Figure-3:**
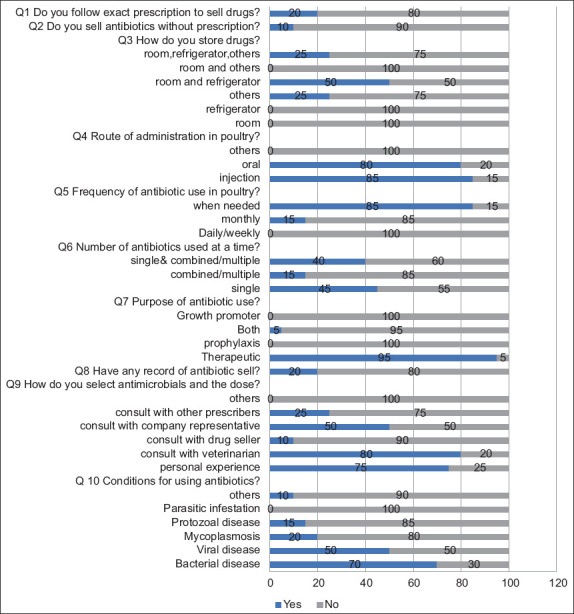
Response on practice of IPs on AMU and AMR. Question 1 (Q1) Do you follow exact prescription to sell drugs? Yes/No. Question 2 (Q2) Do you sell antibiotics without prescription? Yes/No. Question 3 (Q3) How do you store drugs? Yes/No (room, refrigerator, others/room, others/room refrigerator/others/refrigerator/room). Question 4 (Q4) Route of administration in poultry? Yes/No (others/oral/injection). Question 5 (Q 5) Frequency of antibiotic use in poultry? Yes/No (when needed/monthly/daily, weekly). Question 6 (Q6) Number of antibiotics used at a time? Yes/No (single, combined &multiple/combined, multiple/single). Question 7 (Q7) Purpose of antibiotic use? Yes/No (growth promoter/both/prophylaxis/therapeutic). Question 8 (Q8) Have any record of antibiotic sell? Yes/No. Question 9 (Q9) How do you select antimicrobials and the dose? Yes/No (others/consult with other prescribers/consult with company representative/consult with drug seller/consult with veterinarian/personal experience). Question 10 (Q10) Conditions for using antibiotics? Yes/No (others/parasitic/Protozoa/mycoplasmosis/viral/bacterial). IPs=Informal prescribers, AMU=Antimicrobial use, AMR=Antimicrobial resistance.

The questionnaire was pre-tested and divided into sections, such as demographics, which included age, education, occupation, and duration of practice (Table-1). In the questionnaire, six questions were based on knowledge (Figure-1), three on attitude (Figure-2), and 10 questions on the practice of prescribing and selling drugs (Figure-3). The questions were written in English with Bengali translation. The responses were in a “YES” or “NO” format and after the initial recording, they were assigned with scores of “2” and “1” for “YES” and “NO,” respectively.

### Statistical analysis

After sorting the data into frequency tables, percentages, and descriptive statistics, they were transferred to Microsoft Excel and analyzed using Statistical package for the social sciences version 26.0 (IBM Corp., NY, USA). We analyzed the relationship and influence of knowledge on the attitude and practice of IPs. The influence of demographic characteristics on the KAP was also analyzed using two-way analysis of variance, correlation analysis, T-test, and linear regression model test with R-squared (R^2^) analysis. Values with p < 0.05 were considered statistically significant. The data were categorized as correct/incorrect for knowledge, positive/negative for attitude, and good/bad for practice.

## Results

### Sociodemographic characteristics of IPs

The sociodemographic analysis of IPs (Table-1) showed that 12/20 (60%) respondents belonged to the age categories of 25–35 and 55–65 years, whereas 8/20 (40%) respondents made up the middle age categories of 35–45 and 45–55 years. Only 3 (15%) respondents had secondary school education, while 17 (85%) had at least a diploma and above. Furthermore, 7 (35%) of the respondents were veterinary field staff, while 1 (5%) was a compounder, 5 (25%) were quacks, 4 (20%) were drug sellers, and 3 (15%) were medicine company professionals. All of them were included as IPs. Approximately 18 (90%) IPs had over 2 years of experience in selling and prescribing informally, indicating that an overwhelming majority have sufficient experience.

### Informal prescribers’ education by district

District-wise analysis of the educational levels of IPs showed that Jamalpur had the highest number of master’s degree holders; 3 (50%) out of the total 6, while the remaining districts had 1 (16.6%) master’s degree holder each. Sherpur district had 4 (80%) graduates out of five. The respondents from Jamalpur were at least a Secondary School Certificate degree holder.

### Responses on knowledge of IPs on AMU, residues, and AMR

We asked seven questions on the KAP of IPs regarding AMU and AMR. Questions 1, 4, and 6 had sub-questions, to obtain further information based on the main question (Figure-1). Only 75% of IPs had heard about antibiotic resistance and 15% thought antibiotics do not act against bacteria or viruses. Approximately 60% of IPs knew about antibiotic residues.

### Response on attitude of IPs on AMU, residues, and AMR

We asked three questions related to the attitude of IPs on AMU, AMR, and antibiotic residues. Question 3 had sub-questions related to who recommended the sale of antibiotics to them, which reflected the level of self-influence regarding their sales (Figure-2). Approximately 50% of IPs ask farmers to follow a withdrawal period, but most recommend that they complete the medication. Alternatively, only 10% of IPs sell drugs based on a veterinarian’s recommendations.

### Response to practice of IPs on AMU, residues, and AMR

We asked 10 questions regarding the IPs’ practice of AMU and AMR. These questions assessed their role in the sales, precautions taken before selling antimicrobials, the storage of the drugs, and the frequency and type of prescriptions they used (Figure-3). Approximately 20% follow the exact prescription to sell drugs with 85% of them selling antibiotics when needed. Although 40% of IPs simultaneously used single and combined/multiple antibiotics, 70% of them selected the antibiotic and dose based on their personal experience.

### Most frequently used antibiotics by IPs

**Figure-4:**
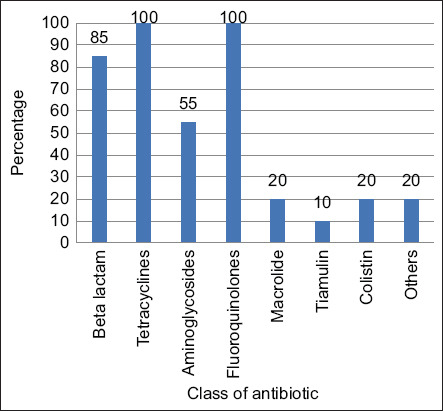
Most frequently used antibiotics by IPs. Percentages of different antibiotic classes used by IPs in the experimental areas. IPs=Informal prescribers.

The most frequently used/prescribed/recommended antibiotics by the IPs were fluoroquinolones and tetracycline (100% frequency), followed by beta-lactam antibiotics (85% frequency) and finally, the aminoglycosides and others. These are important classes of antibiotics with special interest regarding their usage because of the possibility of cross-resistance to humans (Figure-4).

### Correlation of knowledge with attitude and practice on AMU, residues, and AMR by IPs

The results indicated that both age and district of origin significantly influenced the knowledge and attitude of respondents (p < 0.05). Similarly, the results showed that rather than the IPs’ age, their district of origin significantly affected their practice (p < 0.05). Approximately 20% of IPs belong to the age categories 55–65 and 25–35 had the correct knowledge, while 30% had incorrect knowledge. Approximately 25% of IPs from Mymensingh and Sherpur had incorrect knowledge, while 25% from Jamalpur district had correct knowledge. Regarding attitude, 20% of the respondents belonging to the age categories 45–55 and 55–65 had a positive attitude, while 20% of IPs in the age group 25–35 had a negative attitude. For practice, 25% of IPs from Jamalpur and Netrokona had good practice and 25% from Mymensingh had bad practice (Table-2).

**Table-2:** Effect of demographic characteristics on respondents’ knowledge, attitude, and practice regarding AMU, residues, and AMR.

Variable	Knowledge		p-value	Remarks	Attitude		p-value	Remarks	Practice		p-value
		
	Correct n (%)	Incorrect n (%)			Positive n (%)	Negative n (%)			Good n (%)	Bad n (%)	
Age (years)											
25–35	0 (0)	6 (30)	0.025	Sig.	0 (0)	6 (30)	0.010	Sig.	1 (5)	5 (25)	0.130
35–45	2 (10)	1 (5)			2 (10)	1 (5)			2 (10)	1 (5)	
45–55	3 (15)	2 (10)			4 (20)	1 (5)			4 (20)	1 (5)	
55–65	4 (20 )	2 (10)			4 (20)	2 (10)			4 (20)	2 (10)	
Level of education											
SSC	2 (10)	1 (5)	0.796	NS	2 (10)	1 (5)	0.910	NS	2 (10)	1 (5)	0.796
HSC/Diploma	3 (15)	3 (15)			3 (15)	3 (15)			3 (15)	3 (15)	
Graduate	2 (10)	3 (15)			2 (10)	3 (15)			2 (10)	3 (15)	
Masters	2 (10)	4 (20)			3 (15)	3 (15)			4 (20)	2 (10)	
Experience (years)											
1	0 (0)	1 (5)	0.247	NS	0 (0)	1 (5)	0.250	NS	0 (0)	1 (5)	0.247
2	1 (5)	0 (0)			1 (5)	0 (0)			1 (5)	0 (0)	
Others	8 (40)	10 (50)			9 (45)	9 (45)			10 (50)	8 (40)	
Occupation											
Vet. field staff	3 (15)	4 (20)	0.572	NS	3 (15)	4 (20)	0.538	NS	3 (15)	4 (20)	0.572
Compounder	0 (0)	1 (5)			0 (0)	1 (5)			1 (5)	0 (0)	
Quack	2 (10)	3 (15)			2 (10)	3 (15)			2 (10)	3 (15)	
Drug seller	3 (15)	1 (5)			3 (15)	1 (5)			3 (15)	1 (5)	
medicine company professionals	1 (5)	2 (10)			2 (10)	1 (5)			2 (10)	1 (5)	
District											
1	0 (0)	5 (25)	0.001	Sig.	0 (0)	5 (25)	0.001	Sig.	0 (0)	5 (25)	0.001
2	0 (0)	5 (25)			0 (0)	5 (25)			1 (5)	4 (20)	
3	4 (20)	1 (5)			5 (25)	0 (5)			5 (25)	0 (0)	
4	5 (25)	0 (0)			5 (25)	0 (0)			5 (25)	0 (0)	

Level of significance when p ≤ 0.05; computation using SPSS version 26.0. Sig.=Significance, NS=Non significance, AMU=Antimicrobial use, AMR=Antimicrobial resistance

We analyzed the relationship between the respondents’ knowledge and attitude regarding AMU and AMR. Their knowledge (mean ± standard deviation [SD] is 8.60 ± 1.603) and attitude (mean *±* SD is 4.45 ± 0.759) were related with r = 0.805, and the extent of the relationship was R^2^ *=* 0.628 (62.8%). The knowledge significantly influenced the respondents’ attitude regarding AMU and AMR to the extent of 80.5%. The effect of respondents’ knowledge on their attitude regarding AMU and AMR at p *<* 0.05 level of significance F (1, 18) = 33.054, p *<* 0.05, and the extent of the influence (effect) was 80.5%.

Evaluation of the relationship between the respondents’ knowledge and practice of AMU and AMR showed that the knowledge (mean ± SD = 8.6 ± 1.603) and practice (mean ± SD = 16.2 ± 2.726) are related to r = 0.754, and the extent of the relationship was R^2^ *=* 0.545 (54.5%). The respondents’ knowledge significantly affected their practice regarding AMU and AMR with F (1, 18) = 23.736 (p *<* 0.05), and the extent of the influence (effect) was 75.4%. Hence, knowledge significantly contributed to shape the practice of the respondents (farmers) to the extent of 75.4%.

## Discussion

Poultry drug and feed sellers are significantly involved in the misuse and abuse of antimicrobials, which eventually results in AMR. As they are directly involved with the farmers in selling feeds or drugs, they contribute to the development and spread of AMR. This study provides insights into the role of IPs/drugs or feed sellers in the development of AMR, indicating that AMR does not always begin at farms. Many low- and middle-income countries are facing challenges in identifying the routes of emerging threats, such as AMR.

We showed that most IPs (70%) knew about antibiotics and had good knowledge about antibiotic resistance (75%) based on a simple analysis of their responses (Figure-1). However, although 70% knew about the withdrawal period, only half of all IPs recommended adherence to the withdrawal period. This is one of the key issues fueling the transmission of AMR to the consumer since the residues remaining in the edible products are enough to initiate changes in microbial susceptibility.

Surprisingly, our evaluation showed that only 10% of IPs strictly followed the veterinarians’ recommendations regarding antibiotics (Figure-1), indicating that most of them do not consult a veterinarian before selling, even though 80% claimed to do so. Another study similarly reported a high dispensation of poultry drugs without veterinary prescription by pharmacists even though they had moderate knowledge and attitude toward selling poultry antibiotics [11]. Moreover, approximately 70% of IPs used antibiotics against bacterial infection, while 95% used antibiotics only for therapeutic purposes. Furthermore, 45% of IPs use single antibiotics at a time, while the rest use combined or multiple. Further, while 15% of IPs used the drugs monthly, 85% used them when needed. This shows that the respondents did not take much caution before selling or giving advice on the type or frequency of antibiotics to be used by farmers. As they are the first point of call by farmers with relative ease in buying and using antibiotics without any professional restriction, it makes them closer to the farmers. Previously, comparable findings stated that drug sellers significantly influenced AMU, attitude, and practice of poultry farmers as they are mostly educated only to primary levels [12, 13]. We found tetracyclines, fluoroquinolones, beta-lactam antibiotics, and aminoglycosides to have 100%, 100%, 85%, and 55% usage, respectively (Figure-4). Oxytetracycline was to be abused by pharmacists, as reported by Mudenda *et al*. [11]. In rural India, IPs mostly prescribe fluoroquinolones, followed by beta-lactams [14]. This means that the same classes of drugs being overused in the poultry sector are also being used excessively by the public. This can result in resistant infections that are difficult or sometimes impossible to treat due to the overuse of the same drug, causing the microbes to rapidly develop resistance to these drugs.

We found that the age category significantly affected the knowledge and attitude of IPs (P ≤ 0.025) (Table-2). Demographic characteristics also played a significant role in the KAP of respondents in several African countries [15]. However, others observed less knowledge to influence both attitude and practice of respondents related to AMU and AMR [16–19]. Another study in Vietnam showed improper use of antimicrobials among respondents regardless of their educational level, even though they had better knowledge and attitude [20].

We found that the IPs from the 45–55 and 55–65 years age categories had a good attitude compared to the younger age categories. Contrastingly, drug sellers in the younger age group (31–35 and 36–40 years) had good attitudes [16]. We also found that the older age categories (45–55 years and 55–65 years) had better practice but those in the 18–25 and 26–30 years age categories had better practices. Another study in Hungary found that respondents over the age of 35 mostly had a negative attitude toward AMU and AMR in poultry and human applications [21]. Our findings could be because the older age categories are more committed and stable with the business as a permanent source of income, whereas those in the younger age category are still trying to find better or white-collar jobs.

The district of domicile also significantly impacted the knowledge. Surprisingly, IPs from Jamalpur had significantly better knowledge than those from Mymensingh and Sherpur (p ≤ 0.01). The attitude of IPs from Jamalpur and Netrokona also differed significantly (p ≤ 0.001) from that of Mymensingh and Sherpur. The knowledge of IPs influenced their attitude to the extent of 80.5% (R^2^ = 0.628). The knowledge also affected the practice of the IPs with an extent of 75.4% (R^2^ = 0.545). The knowledge of IPs greatly influenced their attitude and practice while the sociodemographics also affected their knowledge and attitude. In Tanzania, a KAP study related to AMU and AMR and its misuse in agriculture, veterinary, and human medicines among communities showed that [10] inadequate knowledge, undesirable attitudes, and bad practices were the major reasons. Other studies have also reported that IPs and other non-professionals are important in dealing with the sales and use of drugs [22, 23].

Due to the nature of collecting information on human behavior using a survey method, this study has several limitations. Each of the four districts in the Mymensingh division, Bangladesh, had a small number of respondents, which could not accurately reflect the state of KAP for IPs. The cause-and-effect relationship between the predictor variables and the dependent binary variables (KAP) of IPs may be influenced by the nature of this cross-sectional study.

## Conclusion

Our results showed that the IPs’ knowledge significantly influenced their attitude and practice. Moreover, their sociodemographics also impacted their knowledge and attitude. However, the knowledge disparity among IPs and other non-professionals in the poultry production sector regarding poultry drug sales and prescription is substantial, resulting in antimicrobial drug misuse.

## Authors’ Contributions

KR and AAS: Conceptualized and managed the project with equal contribution. KR, AAS, SS, and SH: Performed all the experiments and prepared the draft of manuscript. KR, MTH, PI, MSI and FA: Designed and supervised the research, revised, and finalized the draft of manuscript. AAS, UBU, FA, and NS: Performed the statistical analysis and prepared the graphs and tables. KR, MZI, PI, AAS, NS, and FA: Revised the manuscript. All authors have read, reviewed, and approved the final manuscript.
